# Challenges in diagnosis of reninoma: three case reports and literature review

**DOI:** 10.3389/fendo.2026.1799414

**Published:** 2026-07-13

**Authors:** Li Lv, Heye Chen, Baoping Wang, Shuo Li, Bo Bian, Jingqiu Cui, Qing He, Zuoliang Dong, Qiusong Chen, Xin Zhao, Yujie Zhang, Ming Liu

**Affiliations:** 1 Department of Endocrinology and Metabolism, Tianjin Medical University General Hospital, Tianjin, China; 2 Department of Cardiology, Tianjin Medical University General Hospital, Tianjin, China; 3 Clinical Laboratory, Tianjin Medical University General Hospital, Tianjin, China; 4 Department of Imaging, Tianjin Medical University General Hospital, Tianjin, China; 5 Department of Pathology, Tianjin Medical University General Hospital, Tianjin, China

**Keywords:** aldosterone, direct renin concentration (DRC), hypertension, hypokalemia, plasma renin activity (PRA), reninoma

## Abstract

**Background:**

Reninoma is rare and very challenging to diagnose. It is characterized by excessive renin secretion that causes severe hypertension and occurs more frequently in young females. Here, we compare and analyze three cases, which were not initially considered reninoma, because there was no increase in plasma renin activity (PRA), patients had refractory hypertension, or were older.

**Results:**

In two cases, PRA is normal or decreased but the direct renin concentration (DRC) is elevated. The third is in an older man with ambiguous imaging assessments and no evident lateralization of renal vein renin levels.

**Conclusions:**

Our findings suggest that the diagnosis of reninoma should not be completely excluded in patients with no elevated renin activity or refractory hypertension. In addition, a combination of blood tests and imaging analysis is highly recommended, and lateralization of renin levels is more applicable in cases where tumors are relatively small.

## Background

A renal juxtaglomerular cell apparatus (JGA) tumor, also known as a reninoma, is a lesion originating from the renal JGA that leads to excessive production of renin, resulting in secondary hyperaldosteronism, hypertension and hypokalemia. The hypertension caused by reninoma is often resistant to treatment (1–3) and tends to be found in young adults, with a slight female predominance (F:M ratio 1.9:1) and a peak incidence in the second to third decade (4).

**Figure 1:**
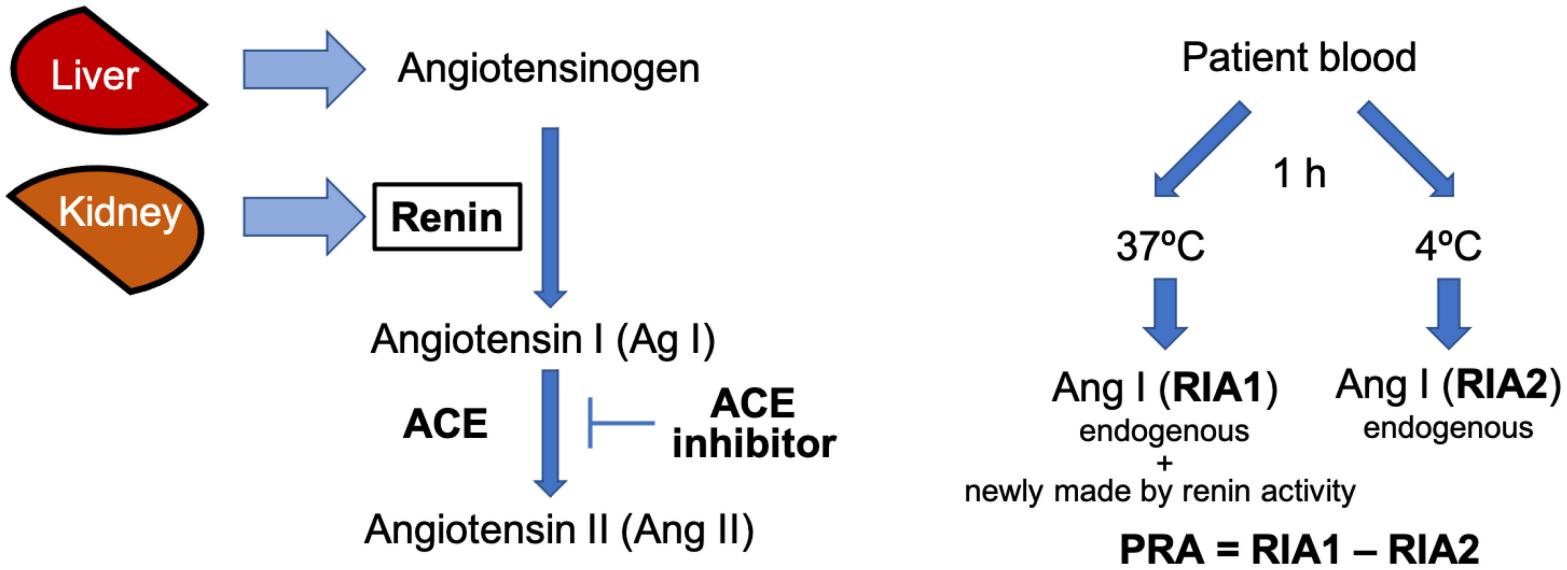
Measurement of PRA.

The first reported case of reninoma was by Robertson et al. in 1967. The diagnosis was made inferentially when a renal neoplasm was found during surgery for bilateral adrenalectomy in a young man with severe hypertension (5). In recent year, ~200 cases have been reported (6) and diagnoses rely on measurements of plasma renin by either its enzymatic activity (plasma renin activity, PRA) or its content (direct renin concentration, DRC). The PRA is measured by radioimmunoassay of the production of angiotensin I (Ang I) from endogenous angiotensinogen per unit of time during incubation at pH 5.7 and 37 °C (7) (**Figure 1**). Normal values are 0.82 ± 0.49 ng Ang I/ml/h in the supine position and 2.37 ± 1.3 ng Ang I/ml/h in the upright position. The plasma concentration of active (post-cleavage) renin can be determined by radioimmunoassay, but DRC is currently commonly determined by automatic chemiluminescence (8).

Here, we report three cases of reninoma. Two of the cases were young women with elevated DRC levels but normal or decreased PRA, and one case is a 45-year-old man. We discuss the clinical manifestations and diagnostic evaluations of this rare disorder. In particular, there may be limitations in the detection of renin activity that account for inaccurate and late diagnoses.

## Case presentation

### Case 1

**Figure 2:**
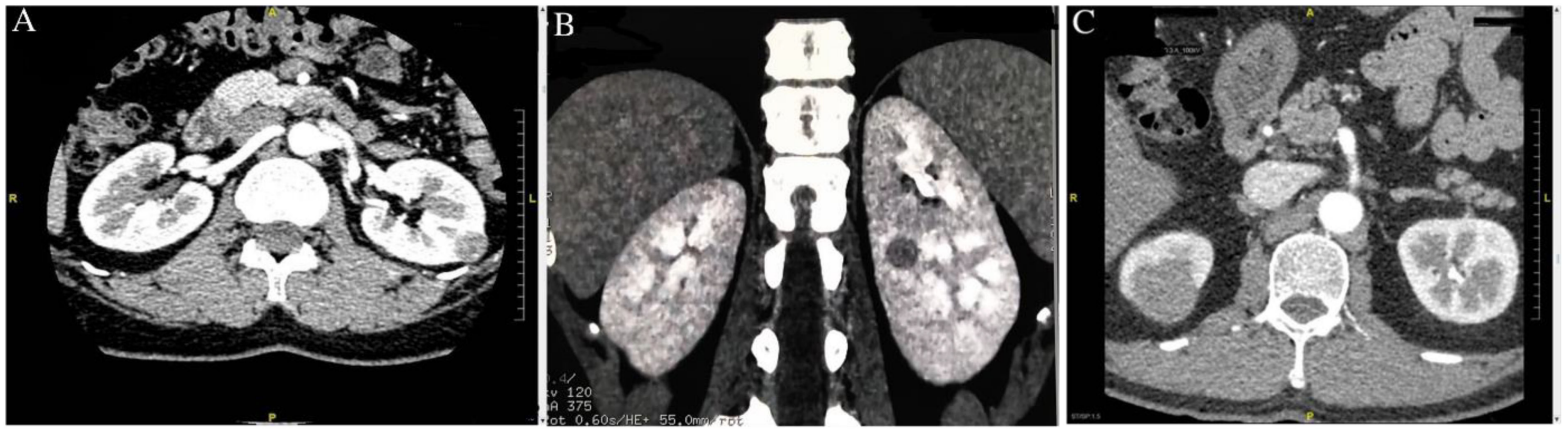
Contrast-enhanced CT of the abdomen. **(A)** Contrast-enhanced CT scan of the abdomen showing a solid nodule (1.5cm diameter) in the lower pole of the left kidney. **(B)** Abdominal contrast-enhanced CT showing a solid, circumscribed, low-density cortical lesion (1.3cm diameter) in the posterior lip of the left kidney. **(C)** Contrast-enhanced CT scan of the abdomen showing a soft tissue mass in the upper pole of the right kidney (3.2cm diameter).

A 28-year-old woman was hospitalized in 2017 with a 10-year history of severe hypertension. In 2007, she was found to have an elevated blood pressure of 180/110 mmHg when she presented with a headache. At this point, the patient is taking amlodipine, enalapril, and metoprolol. Bilateral renal artery computer tomography angiography and bilateral adrenal computed tomography (CT) were performed in 2009 without abnormal findings. In 2013, a decrease in serum potassium (2.8 mmol/L) was noted. Supine and upright PRA were normal and decreased, respectively, whereas plasma aldosterone was elevated (**Table 1**). Abdominal ultrasound and abdominal CT revealed no abnormalities in the bilateral adrenal glands and kidneys. She was suspected of having primary aldosteronism and treated with spironolactone and enalapril. Her blood pressure was 130-190/80–120 mmHg. In 2016, she was re-admitted because of poor blood pressure control. The PRA was still not elevated but plasma aldosterone was increased. Contrast-enhanced CT of the adrenal gland and renal arterial CT angiography showed nodules in the lower pole of the left kidney, with a high possibility of a cyst. Notably, the patient had taken spironolactone and enalapril prior to hospitalization. In 2017, at 28 years of age, the patient was hospitalized for the third time for aldosteronism with “normal adrenal glands” and unruly hypertension. Her blood pressure in the hospital was 240/160 and 220/140 mmHg in the supine and upright positions, respectively. Echocardiography showed left ventricular hypertrophy with mild regurgitation of the mitral, tricuspid, and aortic valves. Creatinine clearance was normal 124 ml/min, though protein excretion was 342 mg/24 h. Her hematological profile was normal except for a hemoglobin level of 157 g/L. Her plasma sodium was 143 mmol/L, potassium 2.6 mmol/L, and serum creatinine 51μmol/L. Urine collection (24h) revealed elevated potassium (78.02 mmol) and decreased sodium (115.2 mmol). Thyroid function and adrenocorticotropic hormone (ACTH) and cortisol levels were within normal limits. Before hospitalization, the patient was taking nifedipine, irbesartan-hydrochlorothiazide, and arotinolol hydrochloride. In 2017, the plasma renin concentration was measured for the first time, as renin activity was measured previously. An elevated DRC was evident in both the supine (241.1μIU/ml; normal range, 2.8–39.9μIU/ml) and upright positions (>500μIU/ml; normal range, 4.4-46.1μIU/ml) with normal plasma aldosterone (ALD) (supine: 7.0ng/dl; normal range, 3.0-23.6ng/dl; upright: 27.4ng/dl; normal range, 3.0-35.3ng/dl; **Table 1**). Abdominal contrast-enhanced CT showed normal adrenal glands; a solid nodule (1.5cm diameter) in the lower pole of the left kidney, with blotch-like density; and slightly delayed imaging after enhancement (**Figure 2A**).

**Table 1:** Test results of the cases.

Case no.	1			2		3
Year	2013	2016	2017	2020.3.18	2020.4.14	2019
Age/Sex	24/F	27/F	28/F	NA	25/F	50/M
Bp/History	190/120, 6	204/144, 6	240/160, 10	NA	137/97, 2	155/110, 10
Serum potassium (mmol/L)	2.8	2.4	2.6	NA	3.8	3.2
24 h urine potassium excretion (mmol/24L)	NA	106.29	78.05	NA	38.9	73.89
Serum creatinine (μmol/L)	NA	60	51	NA	39	57
GFR (mL/min)	NA	115	124	NA	177	132
24 h urine protein excretion (mg/24L)	NA	1148.4	342	NA	31	572
FT3 (pmol/L)	NA	NA	4.65	NA	5.57	4.85
FT4 (pmol/L)	NA	NA	13.72	NA	15.51	11.79
TSH (μIU/mL)	NA	NA	1.079	NA	1.44	10.36
Plasma cortisol (8am) (nmol/L)	NA	NA	16	NA	12.9	16.7
ACTH (8am) (pmol/L)	NA	NA	10.8	NA	12.4	11.6
Supine PRA (ng/ml/h)	0.6^i^	0.09^i^	NA	1.12^ii^	NA	NA
Supine DRC (μIU/mL)	NA	NA	241.1	NA	219.2	>500
Supine Aldosteron (ng/dl)	29.7^n^	58.1^n^	7^nnn^	5.88^nn^	22.5^nnn^	20.4^nnn^
Upright PRA (ng/ml/h)	0.6^i^	0.62^i^	NA	1.45^ii^	NA	NA
Upright DRC (μIU/mL)	NA	NA	>500	NA	437.2	>500
Upright Aldosteron (ng/dl)	39.4^n^	31^n^	27.4^nnn^	8^nn^	32.3^nnn^	44.8^nnn^

Normal range: Serum potassium:3.5-5.5mmol/L; 24h urine potassium excretion: 25.00-100.00mmol/24h; Serum creatinine: 44-115μmol/L; 24h urine protein excretion: 30.0-150.0mg/24h; FT3: 2.43-6.01pmol/L; FT4: 9.01-19.05pmol/L; TSH: μIU/ml; Plasma cortisol(8am): 5.00-25.00ug/dL; ACTH(8am): 0.00-46.00pg/ml; Supine PRA, i:0.05-0.79; ii:0.13-1.74; Upright PRA, i:1.95-3.99; ii:1.45-5; Supine DRC: 2.8-39.9; Upright DRC: 4.4-46.1; Supine Aldosteron, n: 6.0-17.5; nn: 3.0-18.0; nnn: 3.0-23.6; Upright Aldosteron, n: 6.5-30.0; nn: 5.0-31.3; nnn: 3.0-35.3;NA – not available.

**Figure 3:**
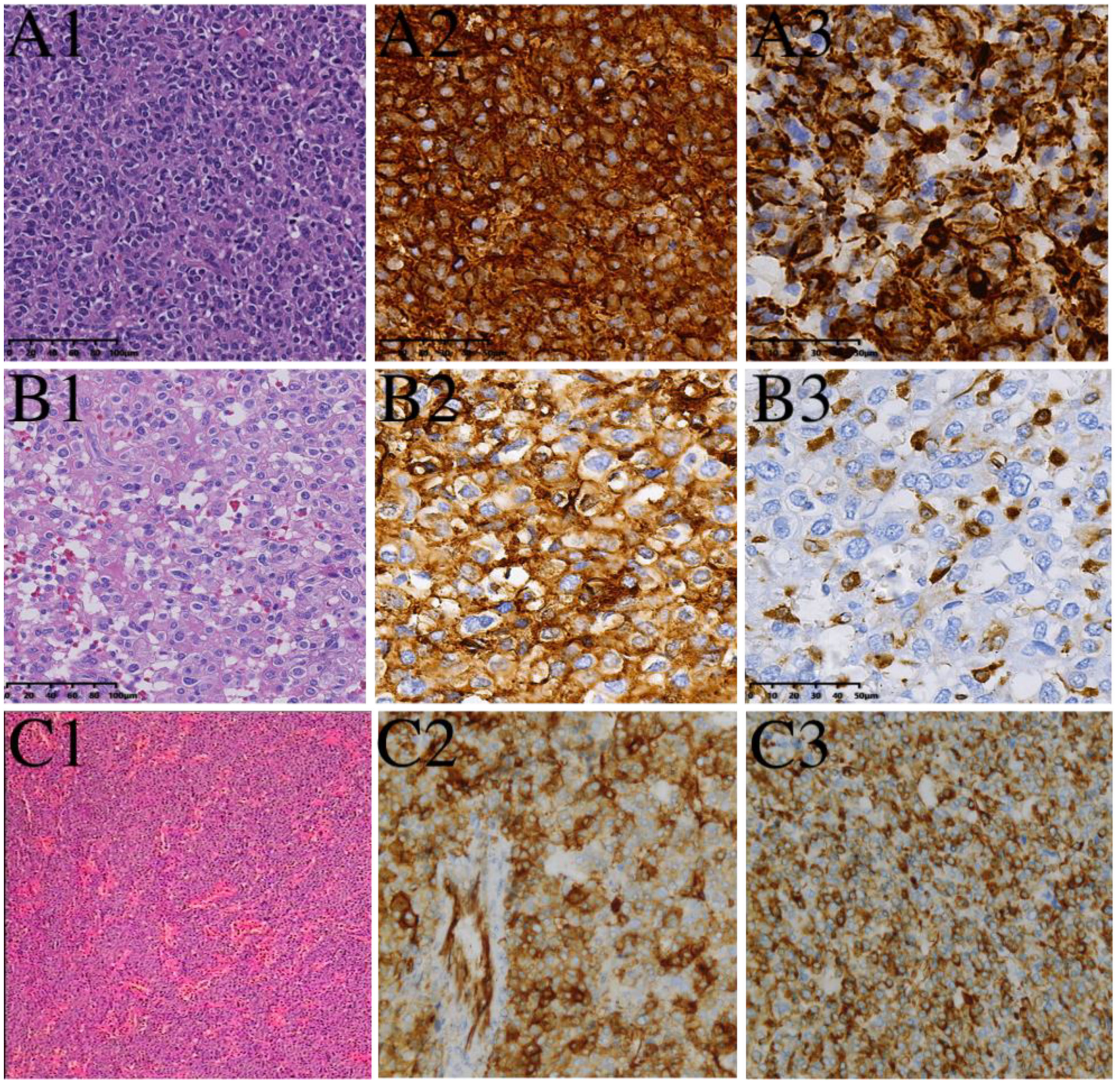
Microscopic findings. The tumor comprises a uniform population of round to polyhedral cells with granular, eosinophilic cytoplasm (**(A1, B1, C1)** ×20, ×10, respectively). The tumor cells stain positive for CD34 (**(A2, B2, C2)** ×40). The tumor cells stain positive for vimentin (**(A3, B3, C3)** ×40).

The patient underwent successful retroperitoneal laparoscopic nephron-sparing tumor enucleation with an uneventful recovery. The tumor was well circumscribed (1.5×1.5×0.8 cm) with pale and dark red sections. Immunohistochemical staining was positive for CD34, vimentin, and actin. No positivity for CK7, EMA, CD10, CA9, ROC, CD117, or CD31 immunostaining was noted (**Figure 3A**). The patient’s blood pressure initially decreased from 190/110 to 102/53 mmHg during the first postoperative hours, but subsequently increased to 130-150/80–100 mmHg. On the 5th postoperative day, her blood pressure was normal (140/80 mmHg) without treatment. Postoperative DRC measurements were also normal: 1.9 μIU/ml, 16.4 μIU/ml, and 4.1 μIU/ml in the supine position on post-surgical days 1, 7, and 14, respectively.

### Case 2

In 2018, a 23-year-old female presented with a borderline elevated blood pressure of 140-150/90–100 mmHg. In 2020, the patient sought help from a local hospital. Laboratory tests revealed normal ALD and PRA in both the supine and upright positions (**Table 1**). The patient was given 2.5 mg amlodipine qd, and her blood pressure was well controlled. She had no family history of hypertension or renal tumor. Upon admission, amlodipine was discontinued to facilitate accurate evaluation of the levels of renin and ALD, and 90 mg diltiazem qd was used instead.

Physical examination revealed a blood pressure of 133/96 mmHg in the right arm and 137/97 mmHg in the left arm in the supine position, with a regular pulse of 72 bpm. Biochemical assay revealed normal liver and renal function, with no hypokalemia (K 3.8 mmol/L). Urinary potassium excretion was 38.9 mmol/24 h. Creatinine clearance was normal 177 ml/min, and protein excretion was 31 mg/24 h. An elevated DRC was evident in both the supine (219.2 μIU/ml; normal range, 2.8–39.9 μIU/ml) and upright positions (437.2 μIU/ml; normal range, 4.4-46.1 μIU/ml) with normal ALD (supine: 22.5 ng/dl; normal range, 3.0-23.6 ng/dl; upright: 32.3 ng/dl; normal range, 3.0-35.3 ng/dl; (**Table 1**). Thyroid hormone, ACTH, and cortisol levels were all within the normal ranges (**Table 1**). Abdominal contrast-enhanced CT showed normal renal arteries and adrenal glands, with a solid, circumscribed, low-density cortical lesion (1.3cm diameter) in the posterior lip of the left kidney, which appeared to be moderately enhanced (**Figure 2B**). There was no obvious enhancement in the arterial phase, but it was progressive in the venous and delayed phases. Magnetic resonance imaging (MRI) plus contrast-enhanced MRI both confirmed the renal cortical lesion. Reninoma was suspected, and selective renal vein sampling (SRVS) was performed. The renal vein renin ratio (RVRR) was 1.4, suggesting lateralization of the renin secretion to the left kidney (**Table 2**).

**Table 2:** Direct renin concentration (DRC) (μIU/ml) during selective renal vein sampling (SRVS).

Case no.	Case 2	Case 3
Peripheral DRC (μIU/ml)	197.8	320.3
Left renal vein DRC (μIU/ml)	258.1	>500
Right renal vein DRC (μIU/ml)	183.2	>500
Ratio, dominant/nondominant side	1.4	NA
Tumor diameter (cm)	1.5×1.3×1.3	3.2×3×3

Retroperitoneal laparoscopic nephron-sparing tumor enucleation was performed in April 2020. The tumor (1.5×1×1cm) was well circumscribed and tan in color. Hematoxylin-eosin staining revealed a neoplasm composed of solid sheets of closely packed polygonal cells with eosinophilic cytoplasm. Immunohistochemical staining was positive for CD34, vimentin, and Syn, with a Ki-67 labeling index of approximately 5%. No positivity for CK7, CD10, 34βE12, EMA, HMB45, SMA, CgA, S-100, or PAX8 immunostaining was noticed (**Figure 3B**). One day after surgery, the patient’s blood pressure normalized to 130/70 mmHg without anti-hypertensive drugs and the PRC decreased from 219.2 μIU/ml to 9.8 μIU/ml. The patient remained normotensive 6 months after surgery.

### Case 3

A 50-year-old man was hospitalized in 2019 for a 10-year history of hypertension. In 2009, the patient’s blood pressure was as high as 155/110 mmHg during the physical examination. In recent years, he needed to take 10 mg amlodipine, 5 mg bisoprolol, and 162.5 mg irbesartan hydrochlorothiazide every day to control his blood pressure at approximately 140/90 mmHg.

The patient’s blood pressure in the hospital was 200/120 mmHg and 190/130 mmHg in the supine and upright positions, respectively. Optic fundi showed Grade I hypertensive changes, and moderate left ventricular hypertrophy was revealed by echocardiography. Plasma potassium was 3.2 mmol/L and urinary potassium excretion 73.89 mmol/24h. Biochemical assay showed normal liver and renal function. Thyroid function and ACTH cortisol levels were within normal limits. Creatinine clearance was 132 ml/min, but protein excretion was 572 mg/24 h. Supine and upright DRC were both >500 μIU/ml, with corresponding plasma aldosterone concentrations of 20.4 and 44.8 pg/dl. Abdominal contrast-enhanced CT showed normal renal arteries and adrenal glands, with a soft tissue mass in the upper pole of the right kidney (3.2cm diameter) with a clear boundary. The enhancement stage was lower than the normal liver parenchyma at each stage. It was considered to be a neoplastic lesion (**Figure 2C**). MRI showed a well-defined isodense lesion 3.2 cm in size in the upper pole of the right kidney. Papillary cell carcinoma was more likely. SRVS was performed to define the function of this lesion. As shown in **Table 2**, the DRC of the left and right renal veins were both above the upper limit of the renin measurement range (**Table 2**).

The patient underwent retroperitoneal laparoscopic nephrosparing tumor excision in March 2019. The tumor was well circumscribed (3.2×3×3cm). Immunohistochemical staining was positive for CD34, vimentin, and CD117, with a Ki-67 labeling index of approximately 3-5%. No positivity for actin, PAX8, WT-1, CD10, CD56, or CD57 immunostaining was noted (**Figure 3C**). One month after surgery, the patient received intermittent metoprolol (25 mg) and amlodipine (2.5–5 mg qd) to control his blood pressure at 120/80 mmHg.

## Discussion

We reported three cases of reninoma diagnosed and treated in our hospital from December 2017 to April 2020. Two of these patients had elevated DRC, but the PRA was in the normal range of the upper limit or reduced and the aldosterone levels were normal or slightly elevated. In one of these two patients, hypokalemia was absent and the blood pressure not significantly elevated, whereas in the other patient, severely elevated blood pressure was associated with left ventricular hypertrophy and retinopathy. RVRR was performed in two of the three cases, with a positive result in one case and no bilateral difference in the other. All three patients underwent abdominal contrast-enhanced CT, in only one case did CT demonstrate a high likelihood of a reninoma, while the other two cases showed renal cysts or renal cell carcinoma. Although the final diagnosis of reninoma was confirmed by pathological results after surgery in all three patients, many difficulties in the diagnosis of reninoma were also confirmed and warrant further discussion.

To date, approximately only 200 cases of reninoma have been reported (9). Hypertension caused by high renin secretion is more common in younger patients, mainly in the second and third decades of life, but the age at diagnosis can vary from 6 to 69 years (6). The patients usually present with severe hypertension and biochemical findings, such as hypokalemia consistent with secondary aldosteronism (6, 10). The most common symptom among patients diagnosed with reninoma is headache (11). Other symptoms include polyuria, polydipsia, nocturia, and myalgia.

Interestingly, in the first two cases, both PRA and DRC were measured, with inconsistent results, with elevated DRC in the absence of elevated PRA though pathological findings confirmed reninoma. Among 43 studies assessing the PRA, the mean PRA was 33.3 ng/ml per h (range 2.8 – 150.9 ng/ml per h) and, in the 40 studies that included normative PRA assay ranges, the mean ± SD PRA was 12 ± 11 times the upper limit of normal (range 1.4 – 58) (6).

What is the reason for the low renin activity in these two cases, which led to the first patient not being diagnosed and treated for up to 10 years? First, the influence of commonly used antihypertensive drugs should be taken into account in detecting the level of renin and aldosterone. Case 1 received amlodipine, enalapril, and metoprolol before being hospitalized for renin activity testing in 2007, and received spironolactone and enalapril before being hospitalized in 2016. With the exception of metoprolol, all of these drugs have the effect of stimulating renin secretion, but the result is that renin activity is not high. Prior to hospitalization in 2017, nifedipine, irbesartan hydrochlorothiazide, and arotinolol hydrochloride were applied; hydrochlorothiazide and arotinolol hydrochloride inhibit renin secretion but result in an increased renin concentration. Therefore, in a renin-secreting tumor, beta blockers have a small effect on renin levels. Case 2 was not treated with any medication prior to renin activity testing and had switched to diltiazem for blood pressure control 2 weeks before the renin test.

Secondly, the renin detection methods are different. The PRA is determined by measuring the production rate of Ang I in plasma; that is, taking two copies of plasma, one of which directly reacts with antibodies to measure the content of Ang I (control tube) and the other is incubated at 37 °C for a certain time and then reacted with antibody to measure the content of Ang I (determination tube). The Ang I content in the determination tube minus that in the control tube, divided by the incubation time, is the production rate of Ang I in a unit of time, which is called the renin activity. The linear range of the test kit used in China at that time was very narrow (0.19–12 ng/ml). The results measured within this range should be reliable, and the results beyond the upper and lower limits are not reliable.

In Case 1, the renin activity detected in 2016 included the values of Ang I measured at 37 °C and 4 °C incubation (upright position: 15.78 and 15.16; supine position: 15.00 and 14.91, respectively), indicating that the renin activity was 0.62 ng/ml per hour in the upright position, lower than the normal range (1.95-4.02 ng/ml per hour), but 0.09 ng/ml per hour in the supine position, within the normal range (0.05-0.79 ng/ml per hour). Thus, the detected Ang I reached a maximum possible reported value, which makes it impossible to distinguish the Ang I concentration at the two temperatures, resulting in a low difference (renin activity) between the two temperatures. The PRA seems low and the aldosterone level was high, making the diagnosis of primary aldosteronism easy. Therefore, when the sample concentration exceeds the detection range, the sample dosage should be increased or the sample should be diluted and tested again. Other considerations include rapid infusion of blood into an anticoagulant tube containing enzyme inhibitors, cooling in an ice water bath, rapid centrifugation, and separation and removal of plasma within 15 minutes after blood collection.

Currently, the DRC is commonly determined by automatic chemiluminescence using monoclonal antibodies to recognize specific epitopes of renin molecules and directly detect the level of active renin in EDTA plasma. The new automated detection method is simple to operate with results in 40 minutes, improved precision, easier standardization, and simultaneous detection of renin and aldosterone in a tube of blood for ARR screening results. Thus, laboratories are beginning to use direct renin concentration as a substitute for renin activity. Unless interfered by female hormones, inappropriate pre-analytical sample processing (such as low-temperature activation), or limited by the bottleneck of the lower limit of quantification in methodology, the change trajectories of PRA and DRC are highly synchronized under the vast majority of physiological and pathological conditions (12). However, it is worth noting that in automated chemiluminescence immunoassay (CLIA) systems, the established standard curve has a fixed dynamic linear range. When the concentration of the target analyte in the sample is extremely high and exceeds the detection limit of the photomultiplier tube, the system can only report a cut-off value (for example, DRC > 500 μIU/ml). Therefore, we recommend performing gradient dilution to determine the true concentration of the target analyte.

In the first two cases, the plasma aldosterone levels were not always elevated. Most cases of reninoma are associated with hyperaldosteronemia and hypokalemia (10, 13, 14). However, some cases have been reported in which aldosterone levels are not elevated (6, 15). Aldosterone levels vary diurnally and are influenced by circulating blood volume, potassium levels, emotional stress, and posture. Case 1 had chronic hypokalemia, which can inhibit the secretion of aldosterone. In case 2, hypokalemia was absent, as was secondary hyperaldosteronism, and her hypertension was easily controlled. This may be related to the functional status of the reninoma. In 58 cases of reninoma that reported with serum potassium data, hypokalemia was detected at presentation in 81% of cases (6). Therefore, hypokalemia is not a necessary condition for the diagnosis of reninoma. The effect of potassium-preserving antihypertensive agents should be excluded when determining serum potassium, as this treatment may obscure the initial effect of secondary hyperaldosteronism on potassium balance.

It is worth noting that different detection methods will affect the accuracy of results. Plasma renin activity (PRA) is an indirect enzyme activity assay that evaluates renin levels by detecting the rate at which endogenous renin cleaves endogenous angiotensinogen to produce angiotensin I (AngI) under specific temperature and pH conditions. Therefore, the detection result of PRA is highly dependent on the substrate concentration of angiotensinogen in blood. In contrast, direct renin concentration (DRC) detection adopts chemiluminescence immunoassay (CLIA), which directly recognizes and binds to the epitope of active renin molecules through specific monoclonal antibodies, thereby determining its true mass concentration. Moreover, the detection of DRC is completely independent of endogenous angiotensinogen levels.

The vast majority of antihypertensive drugs exert systemic interference on the RAAS. By altering sodium load or blocking the negative feedback downstream of angiotensin II, these drugs directly stimulate the kidneys to secrete more active renin. This increase in secretion is reflected in both the absolute amount (elevated DRC) and enzyme activity (elevated PRA) (16). Although there may be subtle differences in the slope of the increase between the two due to discrepancies in detection kinetics, the direction of change is completely consistent. Notably, estrogen-containing oral contraceptives or postmenopausal HRT strongly stimulate hepatic angiotensinogen synthesis (8). When patients undergoing these female hormone regimens receive blood testing, the substantially increased substrate (angiotensinogen) will be rapidly cleaved artificially during *in vitro* incubation, producing excessively high levels of AngI, which consequently leads to a falsely elevated PRA. However, since the actual amount of secreted active renin *in vivo* does not increase (and may even decrease slightly due to the negative feedback of mild volume expansion), the measured DRC will remain normal or lower than normal (17). This is the unique mechanism underlying the severe discrepancy between PRA and DRC measurements induced by female hormones (18). Paired simultaneous PRA and DRC measurements under standardized conditions (same posture, same medication status) were not available in Cases 1 and 2, limiting direct quantitative correlation in these individuals. Due to assay ceiling effects without dilution, absolute renin concentrations in Cases 1 and 3 may have been substantially higher than the reported cut-off values, which reduces the interpretability of lateralization indices.

The use of imaging methods is also one of the key clues to the discovery of reninoma. CT is the most reliable method for tumor localization (19, 20). Unenhanced scans may not detect small isodense lesions or misdiagnose them as cysts; therefore, enhanced scans should be obtained in all cases. The typical manifestations of reninoma are no obvious enhancement in the early arterial phase but delayed enhancement in the venous phase. The degree of enhancement is weaker than that of the surrounding renal parenchyma, which is different from other types of renal tumor (21). MRI is another reliable diagnostic modality (6, 20). In all three cases, imaging diagnoses were made in combination with laboratory tests. Therefore, accurate renin-aldosterone detection is of great significance for imaging diagnosis. The tumor appears as a small avascular area on renal angiography, with false-negative results in up to 42.8%, but it allows for the exclusion of renovascular hypertension due to renal artery stenosis (10). Renal CT arteriography was performed in all three patients and no stenosis was found.

Selective catheterization of the renal veins helps localize the site of renin production (22, 23). An RVRR of 1.2 has been reported to have a sensitivity of 85% and specificity of 75%, whereas an RVRR of 1.5 maximizes specificity while limiting sensitivity (6). Mimran noted that lateralization of renal vein renin levels is more marked in patients with smaller tumors (24). Wong analyzed the tumor size in 86 cases of reninoma utilizing different imaging modalities, finding that the tumor size ranged from 0.2–9 cm in diameter with a mean diameter of 3 cm (6). In case 3, there was no convincing lateralization and the renal tumor was 3.2 cm in diameter, above the average, and in the renal tumor in case 2 was 1.3 cm and the RVRR of 1.4 was diagnostic, supporting Mimran’s hypothesis. In order to get more valuable results, the sample should be diluted and tested again. And it is possible that better lateralization of renin release would be achieved if sampling was performed during captopril stimulation.

The definitive diagnosis is based on histological examination. Grossly, the tumor is well circumscribed and a complete or partial fibrous capsule observed in most cases (4, 25). The cut surface of the tumor is yellow to gray-tan in color with frequent hemorrhage (4). Histologically, the tumor consists of polygonal cells, sometimes with nuclear atypia, and immunohistochemically positive for renin, vimentin, and CD34. However, renin positivity may be observed in some cases of Wilms tumor, renal cell carcinoma (RCC), or renal oncocytoma (26). Genetically, loss of chromosomes 9 and 11 has been frequently observed (27).

## Conclusion

Our three cases confirm that the diagnosis of renin-secreting tumors is not easy because the hypertension may not be severe and laboratory data or imaging studies may not be supportive, making the tumor difficult to find. The failure to recognize such tumors may account for the apparently low prevalence of this disease.

## Data Availability

The raw data supporting the conclusions of this article will be made available by the authors, without undue reservation.
